# Do NGF and LPS Interact Synergistically to Modulate Inflammation in Sheep Endometrial Epithelial Cells?

**DOI:** 10.3390/ijms26146862

**Published:** 2025-07-17

**Authors:** Gabriella Guelfi, Camilla Capaccia, Vicente Francisco Ratto, Cecilia Dall’Aglio, Francesca Mercati, Margherita Maranesi

**Affiliations:** Department of Veterinary Medicine, University of Perugia, Via San Costanzo 4, 06126 Perugia, Italy; camilla.capaccia@dottorandi.unipg.it (C.C.); vicentefrancisco.rattovalderrama@dottorandi.unipg.it (V.F.R.); cecilia.dallaglio@unipg.it (C.D.); francesca.mercati@unipg.it (F.M.); margherita.maranesi@unipg.it (M.M.)

**Keywords:** nerve growth factor, endometrial epithelial cells, TLR4 signaling, prostaglandin biosynthesis, neuroimmune regulation

## Abstract

Neurotrophins and inflammatory mediators are known to influence endometrial function, but their interplay in luminal epithelial cells remains poorly characterized. In this study, sheep endometrial luminal epithelial cells (SELECs) were treated with nerve growth factor (NGF), lipopolysaccharide (LPS), or both, and the effects on gene expression and prostaglandin secretion were evaluated. NGF stimulation alone induced a clear transcriptional activation of *NGF*, neurotrophic receptor tyrosine kinase 1 (*NTRK1*), p75 neurotrophin receptor (*p75NTR*), cyclooxygenase 2 (*COX2*), and steroidogenic acute regulatory protein (*STAR*). LPS treatment selectively increased Toll-like receptor 4 (*TLR4*), *COX2*, and insulin-like growth factor binding protein 6 (*IGFBP6*). Combined NGF and LPS treatment did not enhance the transcriptional response beyond that induced by NGF alone, except for *STAR*. However, co-treatment resulted in a modest increase in prostaglandin production, particularly prostaglandin F2α (PGF2α), but not prostaglandin E2 (PGE2), compared to single treatments, suggesting a possible post-transcriptional modulation rather than a transcriptional synergy. These findings indicate that NGF acts as the primary transcriptional driver in SELECs, while LPS contributes selectively and may enhance prostaglandin output. The observed increase in prostaglandin production may involve post-transcriptional mechanisms, although this remains to be confirmed.

## 1. Introduction

Recent research has redefined the female reproductive tract as not only hormonally responsive but also neuro-immune competent, capable of sensing and integrating local endocrine, immune, and microbial signals [1]. At the core of this dynamic interface lies nerve growth factor (NGF), a prototypical neurotrophin historically associated with neuronal development but now recognized to play a broader role in reproductive physiology.

NGF and its receptors, tropomyosin receptor kinase A (NTRK1/TrkA) and p75 neurotrophin receptor (p75NTR), are widely expressed in reproductive tissues, including the ovary and endometrium. These molecules regulate ovulation, local inflammation, angiogenesis, and steroidogenic activity in both neuronal and epithelial cells [2,3]. NGF also acts as an immunomodulator, upregulating cyclooxygenase 2 (COX2) and promoting prostaglandin synthesis, a mechanism observed in both normal physiology and pathological inflammation [4,5]. Several studies have shown that epithelial and stromal cells in the endometrium express NGF and its receptors under inflammatory conditions, such as endometriosis and chronic pelvic pain disorders. This proinflammatory role of NGF includes increased COX2/PGE2 production and neurogenic sensitization [6,7]. Prostaglandins, particularly prostaglandin E2 (PGE2) and prostaglandin F2α (PGF2α), are key mediators of endometrial immune–endocrine function and are modulated by both inflammatory stimuli and neurotrophins [8,9]. Mechanistically, NGF-driven pathways converge on Nuclear Factor-κB (NF-kB) and extracellular signal-regulated kinases 1/2 (ERK1/2) signaling, facilitating the transcription of genes associated with inflammation [10]. In sheep, a seasonal breeder, NGF expression in reproductive tissues has gained attention due to its dual role in modulating steroidogenesis and inflammatory tone. Maranesi and colleagues have contributed extensively to this field, demonstrating the presence and functionality of NGF systems in the uterine and gonadal tissues of sheep across reproductive phases [11,12,13]. In sheep, endometrial epithelial cells contribute to immune surveillance, prostaglandin production, and local endocrine signaling [13]. In ruminants, additional studies have described NGF involvement in follicular development, corpus luteum function, and endometrial remodeling, highlighting its importance across reproductive compartments. Meanwhile, lipopolysaccharide (LPS)-induced toll-like receptor 4 (TLR4) activation has been associated with altered endometrial function, prostaglandin imbalance, and reduced fertility in both sheep and cattle [14,15].

Despite this growing body of evidence, the specific role of uterine epithelial cells as sensory and regulatory hubs for neuro-immune interactions, particularly under combined exposure to inflammatory stimuli, such as LPS and neurotrophic factors, remains poorly understood. While the interaction between NGF and LPS has been described in immune cell systems [16,17], their combined effects in endometrial epithelial cells remain poorly defined. This gap highlights the need to dissect the molecular and functional responses of endometrial epithelial cells to NGF within a controlled in vitro context. Regardless of extensive evidence of NGF and its receptors in reproductive tissues [3,18,19], the functional impact of NGF on endometrial epithelial cells, particularly under inflammatory challenge, remains unsatisfactorily defined. Most studies have focused on ovarian follicular dynamics [2,20] or immune–endocrine interactions in the stromal compartment [4], overlooking the epithelial layer of the endometrium, which plays a pivotal role in inflammatory sensing, prostaglandin synthesis, and embryo implantation [21,22]. How endometrial epithelial cells integrate NGF and pro-inflammatory signals like LPS, and whether NGF modulates inflammatory pathways such as NF-kB and ERK1/2 via its canonical receptors (NTRK1, p75NTR), remains unaddressed in species-relevant models. Our previous findings in sheep endometrial luminal epithelial cells (SELECs) demonstrated that NGF modulates the expression of both neurotrophic (NTRK1, p75NTR) and innate immune components (TLR4, COX2) [13]. Toll-like receptors (TLRs), particularly TLR4, are critical pattern recognition receptors that mediate the epithelial response to pathogen-associated molecular patterns (PAMPs) via activation of NF-kB and mitogen-activated protein kinase (MAPK) pathways. This research integrates innate immune signaling with local tissue regulation [23].

The present study aims to functionally characterize the molecular and secretory responses of SELEC to combined exposure to NGF and LPS, addressing the currently unresolved role of NGF-driven inflammatory modulation in endometrial epithelial cells. Special attention is given to the regulation by SELECs of key inflammatory and steroidogenic genes such as *COX2*, steroidogenic acute regulatory protein (*STAR*), *TLR4*, and insulin-like growth factor binding protein 6 (*IGFBP6*), as well as to the quantification of prostaglandins PGE2 and PGF2α in the culture medium. The use of NGF, LPS, and a selective NTRK1 inhibitor enables a targeted dissection of NGF signaling pathways, thereby elucidating the autocrine NGF-mediated regulatory activity in SELECs to clarify how neurotrophic and inflammatory signals converge to modulate gene expression and prostaglandin biosynthesis.

## 2. Results

### 2.1. Viability and Epithelial Identity of SELEC

**Figure 1:**
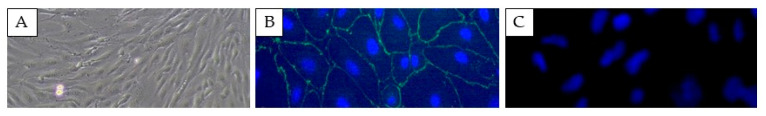
Images of sheep endometrial luminal epithelial cells (SELECs). Phase contrast microscopy shows the characteristic epithelial morphology of SELECs (**A**). Immunofluorescence staining for zonula occludens-1 (ZO-1) in green confirms the presence of tight junctions, with nuclei counterstained with DAPI in blue (**B**). Negative control showing no signal (**C**). Scale bar: 50 μm for all panels.

The MTT colorimetric assay confirmed the high viability of SELECs with percentages of 96% at 12 h. Phase contrast microscopy revealed the typical epithelial morphology of SELECs (Figure 1A). An immunofluorescence analysis for ZO-1 revealed the presence of tight junctions (Figure 1B), further supporting the epithelial identity of SELECs. Negative controls showed no signal, demonstrating the specificity of ZO-1 staining (Figure 1C).

### 2.2. Prostaglandin Secretion Levels in SELEC Culture Medium

**Figure 2:**
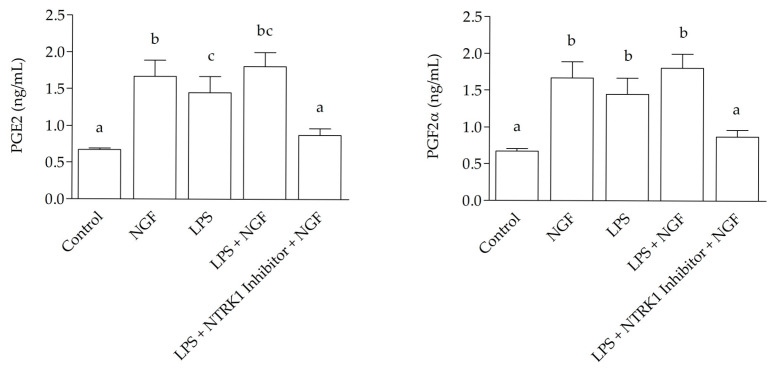
Prostaglandin levels in SELEC medium 12 h after treatment. The graphs show prostaglandin E2 (PGE2, left panels) and prostaglandin F2α (PGF2α, right panels) levels measured by ELISA at 12 h. At 12 h, both PGE2 and PGF2α concentrations were significantly increased in the LPS + NGF group compared to the control and LPS + NTRK1 inhibitor + NGF groups (*p* < 0.0001). Statistically significant differences between groups are indicated by different letters (a, b, c) above the bars.

ELISA results revealed a marked increase (*p* < 0.0001) compared to the control in the concentrations of PGE2 and PGF2α in the culture medium following NGF treatment, with values reaching 1.671 ± 0.125 ng/mL and 1.921 ± 0.45 ng/mL respectively; LPS treatment 1.451 ± 0.155 ng/mL and 1.711 ± 0.058 ng/mL respectively, and an even greater increase after LPS + NGF treatment, with concentrations of 1.806 ± 0.11 ng/mL for PGE2 and 1.859 ± 0.035 ng/mL for PGF2α. These levels were significantly higher than those observed in the control group, which showed 0.672 ± 0.022 ng/mL for PGE2 and 0.517 ± 0.284 ng/mL for PGF2α, as well as in the LPS + NGF + NTRK1 antagonist group, with values of 0.871 ± 0.052 ng/mL for PGE2 and 0.637 ± 0.132 ng/mL for PGF2α (Figure 2).

### 2.3. Gene Expression Signatures in SELECs

The RNA 260/280 ratio was between 1.8 and 2.0, indicating the high purity of the RNA preparation in each sample. Total RNA yield was not significantly different between samples. Minor variations in the total RNA were adjusted RT using a fixed RNA input. The RefFinder tool, which integrates the results of four algorithms—Comparative Delta-Ct, BestKeeper, NormFinder, and GeNorm—identified hypoxanthine-guanine phosphoribosyltransferase 1 (HPRT1) as the most stable endogenous control [24].

**Figure 3:**
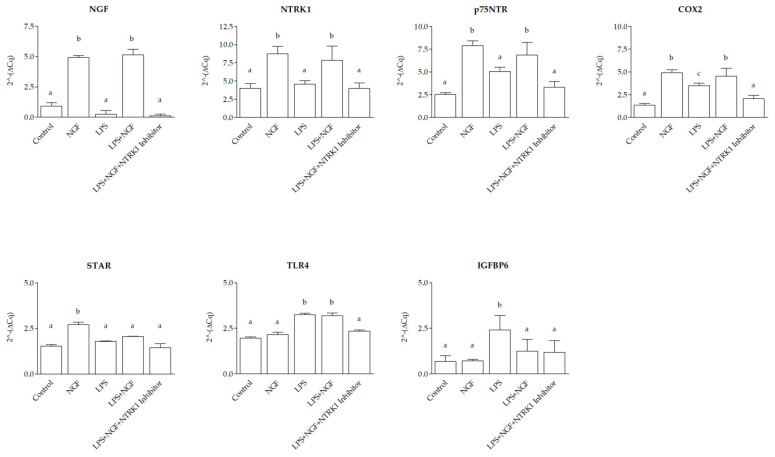
Gene expression modulation in SELEC cells at 12 h following NGF, LPS, and NTRK1 inhibition treatments. The 2^−ΔCq qPCR values for Nerve Growth Factor (*NGF*), neurotrophic receptor tyrosine kinase 1 (*NTRK1*), p75 neurotrophin receptor (*p75NTR*), cyclooxygenase 2 (*COX2*), Toll-like receptor 4 (*TLR4*), and insulin-like growth factor binding protein 6 (*IGFBP6*), and steroidogenic acute regulatory protein (*STAR*) mRNA expression were normalized to the reference gene hypoxanthine-guanine phosphoribosyltransferase 1 (*HPRT1*). With respect to control wells, the addition of NGF induced a significant upregulation of *NGF*, *NTRK1*, *p75NTR*, *COX2*, and *STAR* expression (*p* < 0.0001, *p* < 0.0001, *p* < 0.01, *p* < 0.01, and *p* < 0.01, respectively), whereas *TLR4* and *IGFBP6* expression were not significantly affected (*p* > 0.05 for both). LPS treatment did not significantly alter the expression of *NGF*, *NTRK1*, *p75NTR,* or *STAR* compared to the control (*p* > 0.05 for all) but induced a significant upregulation of *COX2*, *TLR4*, and *IGFBP6* (*p* < 0.05 for *COX2* and *TLR4*; *p* < 0.01 for *IGFBP6*). In all genes analyzed, NGF + LPS treatment resulted in a significant upregulation compared to the control for *NGF*, *COX2*, and *NTRK1* (*p* < 0.0001); *p75NTR* and *TLR4* (*p* < 0.01), and *STAR* and *IGFBP6* (*p* > 0.05). Co-treatment with the NGF + LPS + NTRK1 inhibitor caused a return to basal expression levels in all genes compared to the control (*p* > 0.05 for all). NGF alone induced a significantly higher expression compared to LPS for *NGF* and *NTRK1* (*p* < 0.0001) and for *p75NTR*, *COX2*, and *STAR* (*p* < 0.01). Conversely, *TLR4* and *IGFBP6* expression were significantly higher in LPS-treated than in NGF-treated cells (*p* < 0.01 for both). NGF + LPS treatment differed from NGF alone in the *STAR* gene (*p* < 0.05) and *TLR4* (*p* <0.001). NGF + LPS was significantly different from LPS treatment in all genes (*p* < 0.01) except *STAR* and *TLR4* (*p* > 0.05). The data are presented as mean ± standard deviation. Statistically significant differences between groups are indicated by distinct letters (a, b, c) above each bar.

At 12 h post-treatment, gene expression analysis in SELEC revealed distinct transcriptional responses to NGF, LPS, and their combination, modulated by NTRK1 inhibition. Compared to the control group, transcript levels of *NGF*, *NTRK1*, *p75NTR*, *COX2*, and *STAR* significantly increased following NGF stimulation (*p* < 0.0001, *p* < 0.0001, *p* < 0.01, *p* < 0.01, and *p* < 0.01, respectively), while *TLR4* and *IGFBP6* expression remained unchanged (*p* > 0.05). LPS exposure did not significantly alter *NGF*, *NTRK1*, *p75NTR*, or *STAR* expression (*p* > 0.05) but led to a significant upregulation of *COX2*, *TLR4* (*p* < 0.05), and *IGFBP6* (*p* < 0.01) compared to the control. The combined treatment with NGF and LPS resulted in a significant increase for *NGF*, *NTRK1* (*p* < 0.0001), *COX2* (*p* < 0.01), and *p75NTR* and *TLR4* (*p* < 0.05) compared to the control. In contrast, *STAR* and *IGFBP6* expression remained unchanged (*p* > 0.05). Co-treatment with NGF, LPS, and NTRK1 inhibitor restored expression levels of all genes to baseline (*p* > 0.05 vs. control). When directly compared to LPS alone, NGF treatment induced a stronger upregulation of *NGF*, *NTRK1*, *p75NTR*, *COX2*, and *STAR* (*p* < 0.0001, *p* < 0.0001, *p* < 0.01, *p* < 0.01, and *p* < 0.01, respectively), while *TLR4* and *IGFBP6* expressions were significantly higher in LPS-treated cultures than in those treated with NGF (*p* < 0.01). The combined NGF + LPS treatment differed from NGF alone in the *STAR* gene (*p* < 0.05) and *TLR4* (*p* < 0.001) and from LPS alone in all genes (*p* < 0.01), except *STAR* and *TLR4*, which remained unchanged (*p* > 0.05) (Figure 3).

## 3. Discussion

Our findings demonstrate that NGF stimulation alone significantly upregulates key neuroimmune and steroidogenic genes in SELECs, including *NGF*, *NTRK1*, *p75NTR*, *COX2*, and *STAR*. This transcriptional profile is consistent with previous observations in the endometrium of sheep and rabbits [13], confirming NGF’s central role as a transcriptional regulator in the uterine epithelium. The marked upregulation of *STAR* further underscores the functional connection between epithelial immune activation and steroidogenic capacity, highlighting NGF’s role at the interface between endocrine and inflammatory signaling.

Conversely, LPS, a bacterial ligand that activates TLR4 [25,26], elicited a more selective transcriptional response, significantly increasing *TLR4*, *COX2*, and *IGFBP6* expression. Notably, *IGFBP6* was unaffected by NGF stimulation, reinforcing the idea that its regulation is primarily driven by TLR4 signaling.

The combined treatment with NGF and LPS did not further enhance gene expression beyond NGF alone, except for *STAR*, suggesting a non-additive transcriptional interaction under dual stimulation [16]. Although both NGF and LPS individually modulated inflammatory mediators in SELECs, no synergistic effect was observed when the two stimuli were combined. Several mechanisms may underline this response. First, strong NGF stimulation could activate negative feedback loops that limit further inflammatory gene induction [4]. Second, NGF and LPS may converge on overlapping intracellular (e.g., NF-κB, ERK1/2), potentially leading to pathway saturation or reciprocal interference [17]. Third, NGF may exert context-dependent anti-inflammatory or modulatory effects that attenuate LPS-induced transcription [27]. Lastly, NGF alone may induce near-maximal transcriptional activation of key target genes, resulting in a ceiling effect that prevents further enhancement upon LPS addition [28].

Previous studies in immune cell models have reported mutual amplification: NGF enhanced LPS-induced Interleukin-6 (IL-6) production in peritoneal mast cells [29], and LPS upregulated *NGF* expression via NF-κB in microglia [30]. These findings support the existence of cross-talk in other systems, but our data indicate a modulatory rather than synergistic role of NGF in the epithelial context of sheep endometrium. Further studies are needed to clarify the temporal and mechanistic integration of neurotrophin and innate immune signaling in ruminant reproductive tissues.

The selective increase in PGF2α, but not PGE2, under NGF + LPS co-treatment, despite unchanged gene expression, suggests the involvement of post-transcriptional or enzymatic mechanisms specifically enhancing PGF2α biosynthesis, potentially through increased COX2 activity or preferential substrate channeling toward PGF synthase enzymes. As already mentioned, LPS alone upregulated *IGFBP6* in SELECs. IGFBP6 has been shown to inhibit NF-κB nuclear translocation and suppress inflammatory gene expression. Therefore, its induction by LPS could contribute to the attenuation of NF-κB signaling, thereby limiting pro-inflammatory transcriptional activity. Currently, no direct evidence supports *IGFBP6* transcriptional regulation by NGF or its canonical receptor NTRK1. IGFBP6 is primarily known for its anti-proliferative and immunomodulatory functions and can be regulated by downstream effectors such as MAPK/ERK and NF-κB. Since NGF activates both these pathways via NTRK1, an indirect influence cannot be excluded. However, in our experimental model, TLR4 signaling is the main regulatory route, consistent with prior findings on LPS-dependent IGFBP6 upregulation in endometrial cells [31].

The complete reversal of NGF-induced gene expression and prostaglandin production by NTRK1 antagonism confirms that SELECs primarily rely on canonical NTRK1 signaling. This finding further underscores NGF’s central role as the main upstream regulator of both transcriptional and secretory responses in the endometrial epithelium.

**Figure 4:**
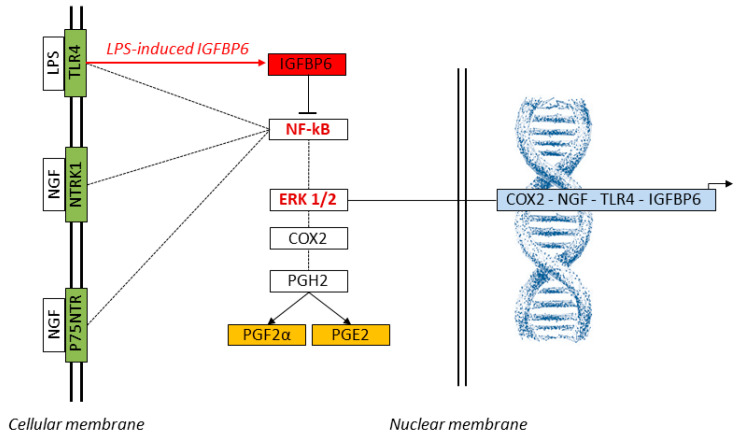
Schematic representation of the signaling network activated by NGF and LPS in SELECs. LPS binds to TLR4, while NGF engages its canonical receptors NTRK1 and p75NTR, activating the intracellular NF-κB and ERK1/2 pathways (KEGG: map04620, map04722). These transcriptional regulators translocate to the nucleus and promote the expression of key inflammatory and neuroimmune genes, including *COX2*, *NGF*, *TLR4*, and *IGFBP6*. The COX2-encoded enzyme catalyzes the conversion of arachidonic acid to Prostaglandin H2 (PGH2), the precursor of PGE2 and PGF2α. The red arrow highlights LPS-induced *IGFBP6* expression, which functions as a negative modulator of NF-κB, potentially contributing to feedback inhibition of inflammatory gene transcription. Genes analyzed by qPCR (*COX2*, *NGF*, *TLR4*, *IGFBP6*) are shown in light blue; prostaglandins measured by ELISA (PGE2, PGF2α) are indicated in orange; membrane receptors assessed by qPCR (TLR4, NTRK1, p75NTR) are in green. Core transcriptional regulators (NF-κB, ERK1/2, IGFBP6) are displayed in red. Dashed lines represent simplified molecular transitions. These interactions suggest that NGF may amplify prostaglandin-mediated tissue remodeling and immune readiness, with implications for implantation and host defense.

Figure 4 illustrates this integrated model of TLR4 and NTRK1 signaling and their downstream regulatory interactions with NF-κB and ERK1/2, highlighting the potential inhibitory role of IGFBP6 in modulating NF-κB activation.

This study has limitations that should be acknowledged. The analysis focused on mRNA expression of selected targets, without complementary assessment at the protein level. While prostaglandin quantification provides functional insight, future studies incorporating protein-level analysis will be instrumental in validating the proposed mechanisms and clarifying the specific contribution of each signaling component. Although NTRK1 inhibition confirms the involvement of the receptor, the downstream effectors, particularly NF-κB and ERK1/2, were not assessed directly. Analyzing their specific contributions and phosphorylation dynamics will require targeted inhibition strategies to define their roles in transcriptional regulation and pathway convergence more precisely.

## 4. Materials and Methods

### 4.1. Animal Selection and Experimental Design

**Figure 5:**
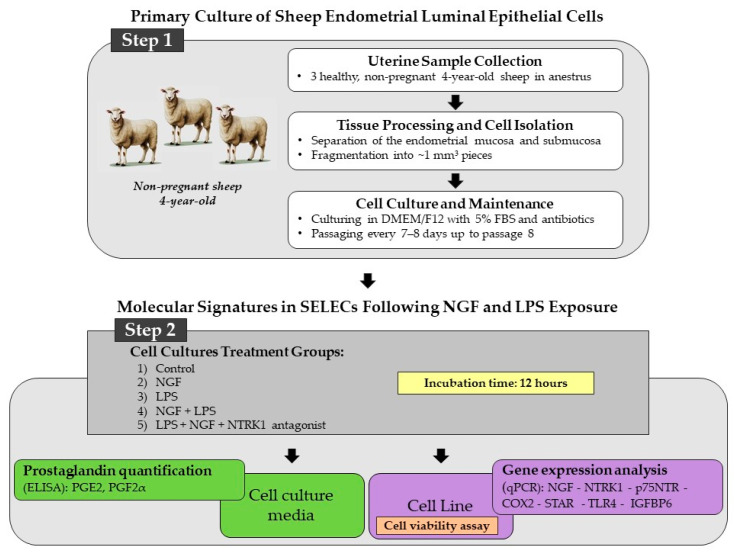
Stepwise overview of the experimental workflow used to investigate NGF-related molecular pathways in SELECs, structured in two main phases. Step 1: SELEC cultures were established from uteri collected from three healthy, non-pregnant, 4-year-old anestrus sheep. Endometrial mucosa and submucosa were separated, fragmented into ~1 mm^3^ pieces, and enzymatically dissociated using 0.1% collagenase type I (Sigma-Aldrich, St. Louis, MO, USA) in DMEM/F12 for 60 min at 37 °C under gentle agitation. After filtration (70 μm mesh) and centrifugation (300× *g*, 10 min), cells were cultured in DMEM/F12 with 5% fetal bovine serum and antibiotics and subcultured every 7–8 days up to passage 8. Step 2: SELECs were exposed for 12 h to five conditions: control, NGF, LPS, NGF + LPS, and NGF + LPS + NTRK1 antagonist. Cell viability (violet box) was assessed by MTT assay. Culture media (green box) were collected for PGE2 and PGF2α quantification via ELISA. Cells (purple box) were processed for total RNA extraction and qPCR analysis of NGF, NTRK1, p75NTR, COX2, STAR, TLR4, and IGFBP6.

Three sheep of the Appenninica breed, raised under semi-extensive conditions and fed a standard diet based on hay and concentrate, were used in this study. The animals were not genetically related and had an average body weight of approximately 65 kg. All subjects were clinically healthy, non-pregnant, and in anestrus, with no signs of reproductive or systemic pathology at the time of slaughter. The uteri were collected from 4-year-old sheep slaughtered for human consumption at a local abattoir according to Council Regulation (EC) No. 1099/2009 on the protection of animals at the time of killing (law n. 333/98, Council Directive 93/119/EC of 22 December 1993) as specified by Annex C of Section II. The experimental procedures were approved by the Ministry of Health (no. of approval: 95/2018-PR). Macroscopic examination of the ovaries revealed the absence of dominant follicles or corpora lutea, confirming the anestrus phase of the sheep. Immediately after collection, approximately 30 uterine fragments per animal were carefully dissected under sterile conditions to establish the SELEC cell line. The experimental workflow, including steps 1 and 2, is illustrated in Figure 5.

### 4.2. Establishment of Primary SELEC Cultures

Uteri were washed with sterile Dulbecco’s phosphate-buffered saline (DPBS), trans-ported to the laboratory, kept at 0 °C, and processed within 2 h of collection to ensure preservation. Endometrial tissue fragments (~1 mm^3^) were washed three times in DPBS containing antibiotics and placed epithelial side down on Petri dishes precoated with 0.1% porcine gelatin. Cells were cultured in DMEM/F12 supplemented with 20% FBS. Once cell growth was established, tissue fragments were removed, and differential plating was performed. Isolated SELEC cells were maintained in DMEM/F12 supplemented with 5% FBS, 2 mM glutamine, and Antibiotic-Antimycotic Solution (1×; containing 100 IU/mL penicillin, 100 µg/mL streptomycin, and 0.25 µg/mL amphotericin B) in a 5% CO_2_ atmosphere at 37 °C. Cells were passaged at a 1:4 ratio every 7–8 days upon reaching 90–95% confluence using trypsin-EDTA for detachment and 10% FBS to inhibit enzymatic activity. SELECs were designated as passage 0 (P0) at initial plating and expanded up to passage 8 (P8) for experimental assays. Details of the reagents used are reported in a previous study by Guelfi et al. (2025) [13].

### 4.3. Immunofluorescence Characterization of Tight Junctions

To confirm the epithelial origin of the cell line, immunofluorescence was performed using the epithelial marker zonula occludens-1 (ZO-1) [32], as described by Guelfi et al. (2025) [13]. Nuclei were counterstained with DAPI. Negative controls, prepared by omitting the primary antibody, were included to verify staining specificity and exclude background fluorescence. Images were acquired using an Eclipse TE200 microscope (Nikon, Tokyo, Japan).

### 4.4. SELEC Incubation with NGF and LPS

At P8, SELECs were seeded in 24-well plates (1 × 10^5^ cells/well) in an F-12/DMEM medium supplemented with antibiotics and incubated at 38 °C in an atmosphere of 95% air and 5% CO_2_. Upon reaching ~80% confluence, cells were treated with either the control medium (100% viability), MEM with 10 μM DMSO to evaluate solvent effects, or a medium with 10% Triton to represent 0% viability, as described by Guelfi et al. (2025) [13]. To assess SELEC functional responses, cells were exposed to five treatment conditions: (1) control (medium alone), (2) NGF (0.36 nM; Miltenyi Biotec, Bologna, Italy), (3) LPS (500 ng/mL in MEM; (Sigma-Aldrich, St. Louis, MO, USA), (4) combined NGF and LPS, and (5) NGF and LPS supplemented with a 10 μM NTRK1 antagonist (Tocris Bioscience, Bristol, UK) diluted in DMSO. Based on our previous findings identifying 12 h as the most informative time point for capturing early prostaglandin-related transcriptional changes, all analyses were conducted at this time point [13]. At the end incubation, cells were harvested for qPCR expression evaluation of *NGF*, *NTRK1*, *p75NTR*, *COX2*, *STAR*, *TLR4*, and *IGFBP6* genes. In parallel, the culture medium was collected for PGE2 and PGF2α quantification by ELISA.

### 4.5. Cell Viability Assay

Cell viability was assessed using the colorimetric MTT cell proliferation assay, which measures mitochondrial activity by the reduction of yellow MTT (3-(4,5-dimethylthiazol-2-yl)-2,5-diphenyltetrazolium bromide) to an insoluble blue formazan product by mitochondrial succinate dehydrogenase. The MTT assay was performed as previously described [13]. A Tecan Infinite M200 spectrophotometer (Tecan Group Ltd., Männedorf, Switzerland) was used to measure the absorbance at 570 nm to calculate cell proliferation, with a reference wavelength of 690 nm. The untreated control group was set as the baseline, corresponding to 100% viability. The intra-assay coefficient of variation (CV) was 5%, and the inter-assay CV, calculated from triplicate measurements, was 6.5%.

### 4.6. Culture Medium: PGE2 and PGF2α Assessment

PGE2 and PGF2α were quantified by ELISA in the culture medium. The kits used and their sensitivity had been previously validated [13]. Prior to analysis, pilot experiments were conducted to determine the optimal dilution of the culture medium. Each plate included culture medium samples and a standard curve, allowing for normalization and inter-plate comparison. All samples were tested in triplicate, and the mean values were used for analysis. The ELISA kits contained plates pre-coated with capture antibodies specific to sheep antigens (PGE2 and PGF2α). A total of 100 μL of culture medium was added to each well and incubated for 120 min at 37 °C. After three washes, 100 μL of avidin-HRP detection antibody was added and incubated for 20 min, followed by three more washes. Subsequently, the substrate solution (90 μL) was added and incubated for 20 min at room temperature. The wells containing the analyte and reagents turned blue. The reaction was stopped with 50 μL of stop solution. Absorbance was measured at 450 nm using a microplate reader (Tecan Spark, Tecan Group Ltd., Männedorf, Switzerland).

### 4.7. SELEC: Gene Expression Signatures

Total RNA was extracted from approximately 500,000 cultured cells using the RNAqueous-Micro Total RNA Kit (Invitrogen, Carlsbad, CA, USA). To remove residual DNA, RNA was treated with DNase I Amplification Grade (Invitrogen, Carlsbad, CA, USA) after extraction. Spectrophotometric (NanoDrop™ 2000/2000c, Thermo Fisher Scientific, Waltham, MA, USA) and fluorometric (Qubit RNA assay, Life Technologies, Carlsbad, CA, USA) measurements were performed to assess RNA quality and quantity. RNA was reverse transcribed to cDNA using SuperScript IV VILO Master Mix (Thermo Fisher Scientific, Waltham, MA, USA) without reverse transcriptase (RT) controls to exclude genomic DNA contamination. To improve the sensitivity of the qPCR analysis, a pre-amplification step was performed using 3 μL cDNA (diluted 1:10), 1 μL TaqMan probes, 10 μL SsoAdvanced™ Preamp Supermix (Bio-Rad, Hercules, CA, USA) and water to a total volume of 20 μL. QPCR amplification was then performed in a final volume of 20 μL using 10 μL SsoAdvanced Universal Probes Supermix (Bio-Rad, Hercules, CA, USA), 1 μL pre-amplification product, and 1 μL TaqMan probes. The probe details are listed in Table 1.

Pre-amplification and QPCR amplification cycling conditions were performed as described by Guelfi et al. (2024) [33] in a 96-well optical plate on the StepOne Plus Real-time qPCR instrument (Applied Biosystems, Carlsbad, CA, USA). Each sample was analyzed in triplicate, and the average quantification cycle (Cq) was calculated using StepOne Software v2.3 (Applied Biosystems, Carlsbad, CA, USA). In parallel, negative controls without the template were analyzed. The algorithms Comparative DeltaCq, BestKeeper, NormFinder, and GeNorm were used to evaluate the expression stability of putative endogenous control genes. Normalization of gene expression data was performed using the 2-ΔCq method [34], where ΔCq target gene = Cq target − Cq endogenous control.

### 4.8. Statistical Analyses

Statistical analyses were performed using Prism software (version 10, GraphPad Software Inc., San Diego, CA, USA). Data were evaluated by one-way ANOVA followed by the Newman–Keuls post hoc test for multiple comparisons. The normality of data distribution was assessed using the Shapiro–Wilk test, and the assumption of homoscedasticity was verified using Levene’s test. These conditions justified the use of parametric analysis. Results are presented as mean ± standard deviation (SD), and statistical significance was defined as *p* < 0.05. All experiments were conducted in at least three independent replicates.

## 5. Conclusions

This study shows that NGF is the main transcriptional driver of inflammatory and steroidogenic responses in sheep endometrial luminal epithelial cells, while LPS exerts selective effects through TLR4 signaling. The lack of transcriptional synergy under NGF and LPS co-treatment suggests a non-additive interaction likely shaped by converging signaling pathways and regulatory constraints. These findings support the role of NGF-driven signaling in modulating endometrial immune–endocrine balance. Understanding how NGF and inflammatory stimuli such as LPS interact in epithelial cells provides new insights into endometrial receptivity, implantation success, and the development of anti-inflammatory strategies in reproductive disorders.

## Figures and Tables

**Table 1:** The table shows the gene acronym and name, TaqMan probe ID, reference sequence, exon boundary, and amplicon length (bp). Target genes are *NGF*, *NTRK1*, *p75NTRCOX2*, *STAR*, *TLR4*, and *IGFBP6*. Putative reference genes (in gray) include *HPRT1*, *TBP*, and *ACTB.* All probes are specific for the species *Ovis aries* (Oa), except NGF, ACTB, and IGFBP6, which are specific for *Canis familiaris* (Cf) with 100% cross-reactivity with sheep.

Gene Symbol	TaqMan ID	Sequence ID	Exon	Bp
NGF (nerve growth factor)	Cf02697134_s1	NM_001194950.1	1	161
NTRK1 (Neurotrophic Receptor Tyrosine Kinase 1)	Oa04767849_g1	XM_027976575.1	14–15	114
p75NTR (p75 Neurotrophin Receptor)	Oa04853013_m1	XM_027974687.1	2–3	62
COX2 (Cyclooxygenase 2)	Oa04657348_g1	NP_001009432.1	4–5	66
STAR (steroidogenic acute regulatory protein)	Oa04657047_m1	NM_001009243.1	4–5	69
TLR4 (toll-like receptor 4)	Oa04656419_m1	NM_001135930.1	1–2	108
IGFBP6 (Insulin-Like Growth Factor Binding Protein 6)	Cf02664455_g1	XP_858473.1	3–4	105
HPRT1 (Hypoxanthine Phosphoribosyltransferase 1)	Oa04825272_gH	XM_015105023.2	7–8	52
TBP (TATA Binding Protein)	Oa04818075_m1	XM_015097549.2	4–5	66
ACTB (Beta-Actin)	Cf04931159_m1	NM_001195845.2	1	52

## Data Availability

The data supporting the findings of this study are included within the article. Further inquiries can be directed to the corresponding authors, Gabriella Guelfi (gabriella.guelfi@unipg.it).
